# Comparative Analysis of High-Intensity versus Low-to-Moderate Intensity Statin Therapy in Patients Undergoing Rotational Atherectomy for Calcified Coronary Artery Disease

**DOI:** 10.3390/life13112232

**Published:** 2023-11-20

**Authors:** Sang-Suk Choi, Jin Jung, Sung-Ho Her, Kyunyeon Kim, Youngmin Kim, Kyusup Lee, Ki-Dong Yoo, Keon-Woong Moon, Donggyu Moon, Su-Nam Lee, Won-Young Jang, Ik-Jun Choi, Jae-Hwan Lee, Jang-Hoon Lee, Sang-Rok Lee, Seung-Whan Lee, Kyeong-Ho Yun, Hyun-Jong Lee

**Affiliations:** 1 Department of Cardiology, St. Vincent’s Hospital, College of Medicine, The Catholic University of Korea, Seoul 16247, Republic of Korea; sader500@naver.com (S.-S.C.); colaking@naver.com (J.J.); gemini729@naver.com (K.K.); ps5231@naver.com (Y.K.); yookd@catholic.ac.kr (K.-D.Y.); cardiomoon@gmail.com (K.-W.M.); babaheesu@gmail.com (D.M.); yellow-night@hanmail.net (S.-N.L.); raph83@naver.com (W.-Y.J.); 2 Department of Cardiology, Daejeon St. Mary’s Hospital, College of Medicine, The Catholic University of Korea, Seoul 34943, Republic of Korea; ajobi7121@gmail.com; 3 Department of Cardiology, Incheon St. Mary’s Hospital, College of Medicine, The Catholic University of Korea, Incheon 21431, Republic of Korea; mrfasthand@catholic.ac.kr; 4 Department of Cardiology in Internal Medicine, Chungnam National University School of Medicine, Chungnam National University Sejong Hospital, Sejong 30099, Republic of Korea; myheart@cnuh.co.kr; 5 Department of Internal Medicine, Kyungpook National University Hospital, Daegu 41944, Republic of Korea; ljhmh75@knu.ac.kr; 6 Department of Cardiology, Jeonbuk National University Hospital, Jeonju 54907, Republic of Korea; 7 Department of Cardiology, Asan Medical Center, University of Ulsan College of Medicine, Seoul 05505, Republic of Korea; seungwlee@amc.seoul.kr; 8 Department of Cardiovascular Medicine, Regional Cardiocerebrovascular Center, Wonkwang University Hospital, Iksan 54538, Republic of Korea; ards7210@wonkwang.ac.kr; 9 Department of Internal Medicine, Sejong General Hospital, Bucheon 14754, Republic of Korea

**Keywords:** statin intensity, rotablation, percutaneous coronary intervention

## Abstract

(1) Background: Moderate-intensity statin therapy, when compared to high-intensity statin therapy in Asian populations, has shown no significant difference in cardiovascular prognosis in small studies. The aim of this study was to compare the prognosis of patients based on statin intensity following rotational atherectomy (RA) during high-complexity percutaneous coronary intervention (PCI). (2) Methods: The ROCK registry, a multicenter retrospective study, included patients who had undergone rotational atherectomy (RA) during percutaneous coronary intervention (PCI) at nine tertiary medical centers in South Korea between January 2010 and October 2019. The patients were divided into high-intensity statin (H-statin) and moderate/low-intensity statin (M/L-statin) therapy groups. The primary endpoint includes outcomes (cardiac death, target vessel myocardial infarction (MI), and target vessel revascularization (TVR)) within an 18-month follow-up period. (3) Results: In this registry, a total of 540 patients with 583 lesions were included. We excluded 39 lesions from the analysis due to the absence of statin usage. The H-statin group had 394 lesions and the M/L-statin group had 150 lesions. There were no significant differences in baseline characteristics, procedural adverse events without heart failure history, triglycerides, or medications between the two groups. The procedural success rate showed a significant difference between the two groups. Multivariate analysis did not show a significant association between M/L-statin therapy and an increased risk of the primary endpoint. In propensity score matching analysis, no significant difference was observed in the primary endpoint either. (4) Conclusions: In high-complex RA PCI, moderate/low-intensity statin therapy is not inferior to high-intensity statin therapy in Korea.

## 1. Introduction

Coronary artery disease (CAD) is a substantial global health issue, and the presence of calcified coronary lesions presents specific challenges in the context of percutaneous coronary intervention (PCI). Advancements in device technology have expanded the indications for percutaneous coronary intervention (PCI) to encompass more complex cases. Furthermore, as the elderly population continues to grow, the probability of encountering patients with heavily calcified coronary artery lesions in clinical settings is on the rise [1]. With these ongoing developments, there is a growing emphasis on techniques for modifying calcified coronary lesions. These methods include balloons, intravascular lithotripsy, orbital atherectomy, and particularly rotational atherectomy (RA) [2,3,4,5]. The RA system features a fast-spinning diamond-coated burr designed to treat calcified lesions. As a result, it serves as an effective tool for modifying plaque within these lesions, ensuring optimal conditions for the insertion of balloons and stents [6,7]. Hence, the use of rotational atherectomy (RA) has emerged as a viable solution for addressing these complex lesions [8,9]. Given that contemporary CAD treatment necessitates a range of methods, including drug-eluting stents (DES) as well as optimal medical management, there is an increasing need to review and update the clinical outcomes of RA when combined with medical treatment in real-world practice. A prior study has compared post-procedural myonecrosis based on the use of pre-procedural statins in patients undergoing rotational atherectomy [10]. However, studies investigating long-term outcomes based on statin intensity in patients with RA have not been reported yet.

We conducted an analysis using data from the Rotational Atherectomy in Calcified Lesions in Korea (ROCK) Registry, which comprised 540 patients undergoing this procedure at nine tertiary centers in Korea from January 2010 to October 2019. These patients were divided into a high-intensity statin group and a low-to-moderate intensity statin group, and we then compared their clinical outcomes. This study represents the first investigation into the effects of varying statin intensities on clinical outcomes of patients with calcified coronary lesions who underwent PCI using RA.

## 2. Materials and Methods

### 2.1. Study Design and Population

**Figure 1:**
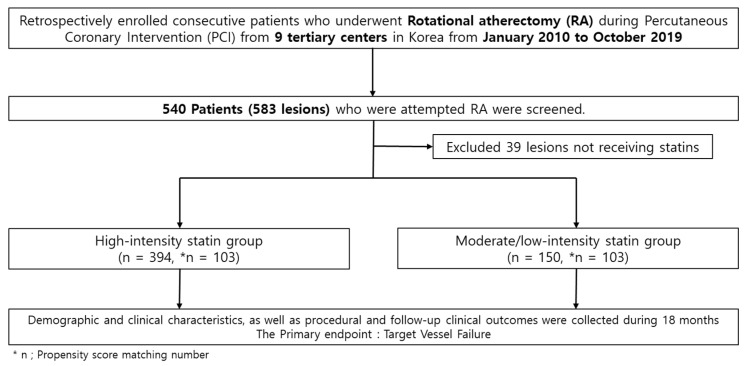
Study population flow chart.

From January 2010 to October 2019, this study enrolled 540 patients (583 lesions) with calcified coronary artery disease (CAD) from the Rotational Atherectomy in the Calcified Lesions in Korea (ROCK) Registry, who underwent PCI using RA at nine tertiary centers in Korea. During this period, patients with consecutive severe calcified coronary lesions and significant stenosis (stenosis ≥ 70% of vessel diameter) who had undergone PCI with RA were retrospectively registered using data from institutional databases. The lesions were categorized into a high-intensity statin group (*n* = 394 lesions) and a low-to-moderate intensity statin group (*n* = 150 lesions). Additionally, there were 39 lesions associated with patients not receiving statins (Figure 1). The ROCK registry did not include the use of ezetimibe or combination agents.

The data were systematically collected at each medical center using a standardized format to document follow-up and procedural details, as well as demographic and clinical characteristics. Follow-up data were gathered based on medical records and thorough consultations with doctors or patients at the time of enrollment during 18 months since the ROCK Registry. This study received approval from the regional ethics committee of each participating hospital. Every patient involved in this study provided written informed consent for the utilization of their clinical data.

### 2.2. RA Procedure

All of the RA procedures were conducted utilizing the Rotablator™ RA system (Boston Scientific, Marlborough, MA, USA) [11]. The treatment strategies, including determination of burr size during the procedure, were executed at the discretion of the treating physician, taking anatomical complexity, patient’s overall condition, and clinical risk factors into careful consideration. Patients who underwent PCI were implanted with drug-eluting stents (DES), primarily second-generation DES, with the exception of one patient who received a first-generation DES.

### 2.3. Definition

According to the 2019 ESC lipid guidelines, the patients were categorized into three groups of statin therapy intensity: low-intensity (simvastatin 10 mg, pravastatin 5–20 mg, lovastatin 20 mg, fluvastatin 20–40 mg, and pitavastatin 1 mg), moderate-intensity (atorvastatin 10–20 mg, rosuvastatin 5–10 mg, simvastatin 20–80 mg, pravastatin 40–80 mg, lovastatin 40 mg, fluvastatin XL 80 mg, and pitavastatin 2–4 mg), and high-intensity (atorvastatin 40–80 mg and rosuvastatin 20–40 mg) statin therapy groups. [12].

The primary clinical outcomes included target vessel failure (TVF), including cardiac death, target vessel spontaneous myocardial infarction (MI), and target vessel revascularization (TVR). The secondary endpoints included all-cause death, cardiac death, MI, stent thrombosis (ST), cerebrovascular accident (CVA), and total bleeding.

Procedural success was defined as the attainment of technical success without incurring in-hospital events or procedural complications, which included in-hospital mortality, cerebrovascular accidents (CVAs) during hospitalization, urgent additional revascularization (CABG or PCI), interventions or surgery for cardiac tamponade, coronary perforation, and procedure-related MI. The definitions of clinical outcomes were the same as those described in a published original article mentioned above [11].

Spontaneous MI was characterized as an elevation in creatine kinase myocardial band or troponin levels beyond the upper limit of the normal range, accompanied by ischemic symptoms or signs during the post-discharge follow-up period. Specifically, spontaneous MI of the target vessel was attributed to the target vessel itself.

TVR was defined as the percutaneous or surgical revascularization of the treated vessel. CVA was characterized as a neurological deficit of central origin lasting more than four hours, confirmed by both imaging and neurologist. All clinical events were validated using source documents gathered from each medical center, and assessed by an independent group of clinicians who were unaware of the type of revascularization procedure.

Bleeding events were characterized based on the thrombolysis in myocardial infarction (TIMI) bleeding criteria. The definition of chronic kidney disease (CKD) was established as an estimated glomerular filtration rate of < 60 mL/min/1.73 m^2^ determined using the modification of diet in renal disease (MDRD) equation based on the initial serum creatinine level [13].

Chronic total occlusion (CTO) was characterized as a lesion with a TIMI grade 0 flow in the occluded segment, and having evidence of occlusion for a minimum of three months. The occlusion duration was estimated by considering the onset of symptoms, the history of angina pectoris, or previous myocardial infarction at the same location, or it was determined through prior angiography. All clinical events were verified using primary records obtained from each hospital, and were independently assessed by a separate team of medical professionals who were blind to the method of revascularization.

### 2.4. Statistical Analysis

Continuous variables are reported as either median and interquartile ranges or as mean ± standard deviation, and were analyzed using Student’s *t*-test. Categorical variables are presented as numbers and percentages, and were compared using either the chi-square test or Fisher’s exact test.

The primary clinical outcomes were evaluated using the Kaplan–Meier method, and compared utilizing the log-rank test. Multivariable Cox regression analyses and propensity matching score analysis were conducted to assess the impact of statin intensity on clinical outcomes. The hazard ratio (HR) and its 95% confidence interval (CI) were also determined. In the multivariate analysis and propensity matching score analysis, the confounding factors were baseline characteristics (age, sex, body mass index (BMI), systolic blood pressure (SBP), heart failure), laboratory parameters (serum albumin, triglycerides), medications (dual antiplatelet therapy (DAPT), beta blocker), and lesion characteristics (intravascular ultrasound sonography (IVUS), procedure success) (Table 1, Table 2 and Table 3).

The clinical event rates were calculated using the Kaplan–Meier method in time-to-first-event analyses based on propensity matching scores, and comparisons were made using the log-rank test. For subgroup analysis, Cox regression analysis was performed and visualized using forest plots. A *p*-value of less than 0.05 was regarded as statistically significant. All statistical analyses were performed using Statistical Analysis Software (SAS) version 9.2 (SAS Institute, Cary, NC, USA).

## 3. Results

### 3.1. Baseline Characteristics

Table 1 provides a comparison of baseline characteristics between high-intensity statins (H-statin, *n* = 394) and moderate- or low-intensity statins (L–M statin, *n* = 150) groups. The mean age was 71.9 years for the H-statin group, while it was 70.1 years for the L–M statin group. The procedure complexity was primarily categorized as B2 and C (92.9% vs. 90.7%). There were no statistically significant differences in age, gender, history of smoking, prevalence of DM, hypertension (HBP), CKD, prior percutaneous coronary intervention (PCI), coronary artery bypass graft (CABG), myocardial infarction (MI), or procedural complexity between the two groups. However, there were statistically significant differences in the utilization of intravascular ultrasound (IVUS), history of heart failure, triglycerides (TG), and medications (dual antiplatelet therapy, beta blocker therapy). The procedural success rate was 97.7% in the H-statin group and 93.3% in the L–M statin group, showing a significant (*p* = 0.013) difference. It is worth noting that all of the baseline characteristics showed no significant differences between the two groups after propensity matching.

### 3.2. Clinical Outcomes

During a median follow-up duration of 18 months, no significant differences was found in the primary outcome, target vessel failure (TVF), between the high-intensity statin (H-statin) group and the moderate/low-intensity statin (M/L-statin) group (11.2% vs. 12.7%, log-rank *p* = 0.501 by multivariate analysis; 18.5% vs. 11.7%, log-rank *p* = 0.266 by propensity matching score analysis). The secondary outcomes also did not show significant differences (cardiac death: 5.1% vs. 2.7%, log-rank *p* = 0.247 by multivariate analysis, 8.7% vs. 2.9%, log-rank *p* = 0.091 by propensity matching score analysis; target vessel myocardial infarction: 1.3% vs. 1.3%, log-rank *p* = 0.921 by multivariate analysis, 1.0% vs. 0.0%, log-rank *p* = 0.323 by propensity matching score analysis; target vessel revascularization: 6.4% vs. 8.7%, log-rank *p* = 0.283 by multivariate analysis, 10.7% vs. 7.8%, log-rank *p* = 0.566 by propensity matching score analysis; cerebrovascular accident (CVA): 1.5% vs. 2.7%, log-rank *p* = 0.369 by multivariate analysis 1.0% vs. 0.0%, log-rank *p* = 0.551 by propensity matching score analysis; stent thrombosis: 0.8% vs. 1.3%, log-rank *p* = 0.525 by multivariate analysis, 1.0% vs. 1.0%, log-rank *p* = 0.970 by propensity matching score analysis; total bleeding: 5.3% vs. 6.0%, log-rank *p* = 0.724 by multivariate analysis, 3.9% vs. 5.8%, log-rank *p* = 0.468 by propensity matching score analysis) (Table 4).

**Figure 2:**
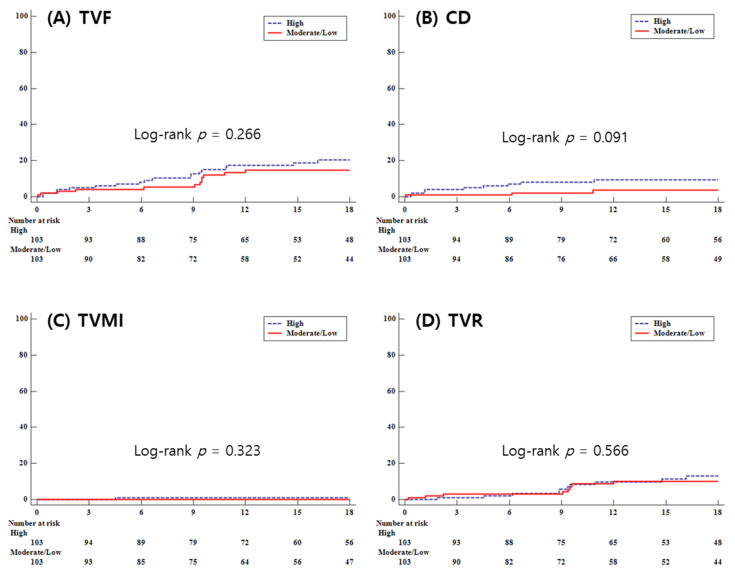
Kaplan–Meier curves representing clinical outcomes during the follow-up period. (**A**) TVF, target vessel failure; (**B**) CD, cardiac death; (**C**) TVMI, target vessel myocardial infarction; (**D**) TVR, target vessel revascularization.

Figure 2 presents the Kaplan–Meier curves for clinical outcomes during follow-up for both groups, showing no significant differences in TVF, CD, target vessel myocardial infarction (TVMI), or TVR.

**Figure 3:**
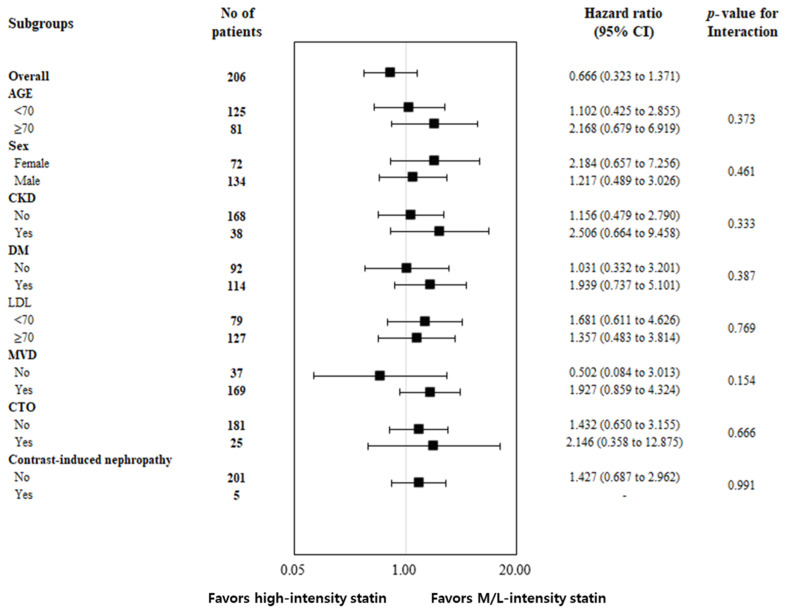
Forest plot for subgroup analysis comparing the incidence of target vessel failure between the high-Intensity and moderate/mow-intensity statin groups.

In a forest plot sub-analysis that compared the two groups based on an age of 70 years, gender, the presence of CKD, DM, a low-density lipoprotein (LDL) level of 70, the presence of multivessel disease (MVD), CTO, and contrast-induced nephropathy (CIN), no significant differences were observed between the two groups (Figure 3).

## 4. Discussion

The main findings of this study indicate that the M/L-statin group is not significantly associated with an increased risk of the primary end point (cardiac death, target vessel MI, and TVR) during the 18-month follow-up period through multivariate analysis and propensity-score matching analysis when compared to the H-statin group.

The ‘ROtational atherectomy in Calcified lesions in Korea (ROCK)’ registry in our study represents the largest multicenter registry with patients receiving RA in Korea. In our registry, patients underwent PCI using drug-eluting stents (DESs), particularly second-generation DES, with the exception of one patient with a first-generation DES, which reflects the RA clinical outcomes in the current revascularization strategy for significant CAD [14,15]. Recognizing that the current treatment of CAD requires not just multiple techniques like drug-eluting stents (DES), but also the best optimal medical treatment, it is essential to consider improved medical treatment options. This study aimed to investigate the relationship between statin intensity, as a component of medical treatment, administered to patients who underwent RA for highly calcified lesions, and their subsequent prognosis, with a particular focus on adverse events.

Multiple studies have provided evidence supporting the use of statins for lipid-lowering purposes in reducing mortality rates in patients with coronary artery disease, both in primary and secondary prevention settings [15,16,17,18,19,20]. The favorable effects of statins are linked to their pleiotropic effects with anti-inflammatory activity, improvement in endothelial function, reduction in oxidative stress, and antithrombotic activity [21,22]. In an animal model with myocardial ischemia, statins have been shown to reduce myocardial injury and stimulate nitric oxide production, showing some direct vascular and cardioprotective effects of statins [23]. Moreover, research has indicated that statins may have the potential to exert protective effects directly within myocytes, suggesting that myocytes themselves may serve as both initiators and responders to the pleiotropic effects of statins [24]. This finding does not rule out the hypothesis of vascular protective effects associated with the cholesterol-independent effects of statins. Statins provide protection, not only to the endothelial cells lining blood vessels, but also to myocytes, indicating a complex interplay between these cells within an in vivo environment. Statins have shown the potential to reduce coronary atheroma and decrease cardiovascular events [25]. Furthermore, previous research studies have indicated that statin-mediated atheroma calcification, the procalcific effect of statins, is linked to beneficial effects on plaque stability [26,27]. In this study, researchers conducted a comparative analysis of changes in coronary atheroma volume and calcium indices among three distinct patient groups: high-intensity statin therapy, low-intensity stain therapy, and those without statin therapy. Using a post hoc analysis approach with eight prospective randomized studies utilizing serial coronary intravascular ultrasound, the researchers observed sequential modifications in coronary percent atheroma volume and calcium indices within matched coronary segments of individuals diagnosed with coronary artery disease. This study suggests that statins, in addition to their plaque-regressive effects, may also promote coronary atheroma calcification, revealing another potential mechanism by plaque stabilization.

As a result of these statin effects, the guidelines for high-risk procedures recommend the use of high-intensity statins rather than low- to moderate-intensity statins [12]. Since these guidelines were not specifically tailored to the Asian population, and there was no evidence in Asian populations demonstrating clinical benefits of using high-intensity statins over low-intensity statins, concerns about the adverse effects of high-intensity statins have led to hesitancy in prescribing statins, even among high-risk Asian populations. In Japan, A clinical trial known as the REAL-CAD trial [Randomized Evaluation of Aggressive or Moderate Lipid Lowering Therapy with Pitavastatin in Coronary Artery Disease], a prospective multicenter trial with high-risk patients randomly allocated to receive pitavastatin at a daily dosage of either 1mg or 4mg, demonstrated that high-intensity statins did not show serious adverse events, and were correlated with a lower risk of atherosclerotic cardiovascular disease (ASCVD) events when compared to low-intensity statins [28].

However, subsequent studies, including research involving Asian populations, have indicated no differences in clinical outcomes based on statin intensity [29,30,31]. One of these studies was conducted among Korean patients who underwent PCI with DES for angina between 2011 and 2015. It compared high-intensity statin therapy groups with moderate-intensity statin therapy groups using a propensity score matching analysis [30]. This study found no significant difference in the primary endpoint, including all-cause death and myocardial infarction. This suggests that moderate-intensity statin therapy could be considered as an initial treatment strategy with similar clinical efficacy when compared to high-intensity statin therapy in Asian patients with angina undergoing PCI. This study represents the first attempt to compare prognoses based on statin intensity in patients with a highly calcified lesion using rotational atherectomy.

In this study, no significant differences were observed in the primary and secondary clinical outcomes between the high-intensity statin and moderate/low-intensity statin groups. This suggests that high-intensity statin therapy may not necessarily provide additional benefits compared to moderate/low-intensity statin therapy in terms of reducing adverse events.

The reasons why high-intensity statin therapy did not demonstrate better clinical outcomes in this study include the following. Firstly, these results can be interpreted in the light of previous findings that highlight pharmacokinetic variations among different racial groups. While it is recognized that higher-intensity statin therapy leads to improved clinical outcomes in Western populations [32,33], guidelines established for statin therapy may not be directly relevant to Asian patients, as their clinical and genetic profiles differ from those of Western populations [34,35]. Furthermore, a pharmacokinetic study has proposed that the varying effectiveness of statins in specific populations may be linked to differences in statin pharmacokinetics between East Asian and Western patients [36]. In this study involving rosuvastatin, it was observed that plasma exposure and its metabolites were significantly higher in an Asian population when compared to a white population in the same environment. Based on the results of these studies, a prior study has proposed that low- to moderate-intensity statin therapy is sufficient for Koreans [37], consistent with findings of the present study.

Secondly, we focused on paradoxical procalcific effects of statins [38]. Cholesterol-rich plaques are susceptible to rupture [39]. It is known that statins can lead to increased calcification in atheroma found within coronary arteries [40]. It has been proposed that statins may contribute to convert cholesterol-rich plaques to stable calcified plaques, thereby promoting the stabilization of soft tissues [26]. Rotablation is commonly performed in cases involving severe calcific lesions. Patients who undergo this procedure typically exhibit a greater accumulation of calcium in their vessels compared to those undergoing other PCI treatments without RA, irrespective of statin use. Our study included patients with advanced coronary calcification and a high burden of comorbidities. Diabetes mellitus was present in over half of the study population, and multivessel diseases were observed in more than 80% of the patients. Hence, in patients who already have a substantial amount of calcium, the necessary calcium for atheroma stabilization may already be present in the coronary arteries, explaining the lack of differences found between the M/L-statin group and H-statin group. This suggests that, irrespective of whether the statin dosage is high or low, it may not play a significant role in stabilizing lesions that are already extensively calcified. This contrasts to the total calcification score measured by computed tomography (CT) traditionally used to predict the prognosis of CAD [41,42].

Our study has some limitations. Firstly, this study was retrospective and not conducted randomly. However, our study showed little selection bias except for medications and IVUS. Secondly, the proportion of participants in the M/L-statin group was relatively small, and the number analyzed for propensity score matching was also low at 103. Therefore, caution is required in interpreting the results. Thirdly, after statin therapy, we were unable to confirm any improvements in LDL levels, the status of statin dose change after discharge, or inflammatory markers such as CRP between high-intensity and non-high-intensity groups. Finally, we could not account for potential variables of adverse events from statin use other than MACE in our research.

This study is the first to compare clinical outcomes based on statin intensity among patients who underwent rotational atherectomy for calcified coronary arteries. No significant difference in the clinical outcomes was observed based on statin intensity between the two groups. However, as discussed earlier, further research should aim to optimize and individualize statin intensity decisions for calcified coronary artery lesions by considering factors such as race, underlying chronic diseases (ESRD, DM, etc.), and the nature of the calcified lesions [12].

## 5. Conclusions

In high complex RA PCI, moderate/low-intensity statin therapy is not inferior to high-intensity statin therapy in Korea.

## Figures and Tables

**Table 1:** Baseline characteristics.

	Overall (*n* = 544)			Propensity Score Matching Analysis		
	High	Moderate/Low		High	Moderate/Low	
	*n* = 394	*n* = 150	*p*-Value	*n* = 103	*n* = 103	*p*-Value
Age, years	71.9 ± 9.7	70.1 ± 11.0	0.067	71.6 ± 8.3	70.6 ± 11.3	0.453
Male (%)	232 (58.9)	100 (66.7)	0.096	70 (68.0)	64 (62.1)	0.461
BMI (kg/m²)	24.0 ± 3.7	24.8 ± 4.3	0.059	24.7 ± 3.9	24.6 ± 4.4	0.906
SBP (mmHg)	133.7 ± 24.3	128.9 ± 21.3	0.034	130.3 ± 20.7	129.0 ± 21.2	0.615
DBP (mmHg)	74.6 ± 12.7	74.4 ± 12.1	0.862	74.1 ± 12.0	75.2 ± 12.8	0.515
Smoking (%)	76 (19.3)	29 (19.3)	0.991	22 (21.4)	21 (20.4)	>0.999
HTN (%)	311 (78.9)	115 (76.7)	0.566	86 (83.5)	76 (73.8)	0.121
DM (%)	227 (57.6)	84 (56.0)	0.734	58( 56.3)	56 (54.4)	0.892
Hyperlipidemia (%)	172 (43.7)	76 (50.7)	0.142	37 (35.9)	52 (50.5)	0.053
CKD (%)	72 (18.3)	24 (16.0)	0.534	20 (19.4)	18 (17.5)	0.864
Dialysis (%)	34 (8.6)	15 (10.0)	0.618	9 (8.7)	11 (10.7)	0.824
Previous PCI (%)	101 (25.6)	43 (28.7)	0.474	35 (34.0)	29 (28.2)	0.451
Previous CABG (%)	16 (4.1)	10 (6.7)	0.203	4 (3.9)	7 (6.8)	0.549
Previous MI (%)	49( 12.4)	15 (10.0)	0.431	9 (8.7)	10 (9.7)	>0.999
CVA (%)	49( 12.4)	25 (16.7)	0.198	11 (10.7)	15 (14.6)	0.524
PVD (%)	27 (6.9)	10 (6.7)	0.939	11 (10.7)	7 (6.8)	0.481
Chronic lung disease (%)	27 (6.9)	9 (6.0)	0.721	8 (7.8)	6 (5.8)	0.791
Heart failure (%)	50( 12.7)	29 (19.3)	0.049	22 (21.4)	20 (19.4)	0.856
LV_EF (%)	53.0 ± 13.5	52.7 ± 13.6	0.810	51.9 ± 14.2	52.7 ± 14.0	0.700
Atrial_fibrillation (%)	40 (10.2)	10 (6.7)	0.209	16 (15.5)	6 (5.8)	0.053
Clinical_diagnosis						
Stable angina (%)	144 (36.6)	38 (25.3)	0.175	32 (31.1)	30 (29.1)	0.510
Unstable angina (%)	121 (30.7)	51 (34.0)		36 (35.0)	30 (29.1)	
NSTEMI (%)	89 (22.6)	44 (29.3)		28 (27.2)	34 (33.0)	
STEMI (%)	12 (3.1)	7 (4.7)		3 (2.9)	5 (4.9)	
Silent ischemia (%)	27 (6.9)	10 (6.7)		4 (3.9)	4 (3.9)	
DCMP/ICMP (%)	1 (0.3)	0 (0.0)		0 (0.0)	0 (0.0)	

BMI, body mass index; SBP, systolic blood pressure; DBP, diastolic blood pressure; HTN, hypertension; DM, diabetes mellitus; CKD, chronic kidney disease; PCI, percutaneous coronary intervention; CABG, coronary artery bypass graft; MI, myocardial infarction; CVA, cerebrovascular accident; PVD, peripheral vascular disease; LV_EF, left ventricle ejection fraction; NSTEMI, non-ST segmental elevation myocardial infarction; STEMI, ST segmental elevation myocardial infarction; DCMP, dilated cardiomyopathy; ICMP, ischemic cardiomyopathy.

**Table 2:** Laboratory analyses and medications.

	Overall (*n* = 544)			Propensity Score Matching Analysis		
	High	Moderate/Low		High	Moderate/Low	
	*n* = 394	*n* = 150	*p*-Value	*n* = 103	*n* = 103	*p*-Value
Hb	12.3 ± 1.8	12.5 ± 1.9	0.275	12.6 ± 1.8	12.5 ± 1.9	0.544
Triglycerides	115.8 ± 71.6	132.6 ± 86.0	0.045	125.3 ± 81.5	122.1 ± 77.9	0.740
Total cholesterol	141.8 ± 37.3	146.1 ± 39.8	0.245	143.5 ± 40.5	147.6 ± 41.7	0.427
LDL cholesterol	83.0 ± 40.0	87.3 ± 36.9	0.284	86.0 ± 56.5	88.1 ± 39.9	0.748
HDL cholesterol	46.3 ± 13.3	45.6 ± 16.1	0.636	45.9 ± 14.9	44.5 ± 13.1	0.466
hsCRP	3.6 ± 13.7	2.8 ± 7.0	0.180	6.9 ± 14.9	6.1 ± 8.1	0.652
	0.1 (0.3–1.7)	0.2 (0.4–1.8)		1.7 (0.1–8.2)	1.9 (0.3–11.2)	
HbA1c	6.7 ± 1.3	6.7 ± 1.4	0.954	6.6 ± 1.4	6.6 ± 1.5	0.738
NOAC	11 (2.8)	6 (4.0)	0.581	3 (2.9)	3 (2.9)	>0.999
DAPT	380 (96.5)	150 (100.0)	0.014	103 (100.0)	103 (100.0)	>0.999
Aspirin	388 (98.5)	150 (100.0)	0.195	102 (99.0)	103 (100.0)	>0.999
P2Y12 inhibitor	391 (99.2)	150 (100.0)	0.565	102 (99.0)	103 (100.0)	>0.999
Cilostazol	51 (12.9)	23 (15.3)	0.468	11 (10.7)	7 (6.8)	0.481
Beta blocker	292 (74.1)	98 (65.3)	0.042	67 (65.1)	75 (72.8)	0.215
ACEi/ARB	253 (64.2)	97 (64.7)	0.921	64 (62.1)	63 (61.2)	>0.999

Hb, hemoglobin; DAPT, dual antiplatelet therapy; NOAC, new oral anticoagulant; ACEI/ARB, angiotensin-converting enzyme inhibitor/angiotensin II receptor blocker.

**Table 3:** Lesion and procedural characteristics.

	Overall (*n* = 544)			Propensity Score Matching Analysis		
	High	Moderate/Low		High	Moderate/Low	
	*n* = 394	*n* = 150	*p*-Value	*n* = 103	*n* = 103	*p*-Value
Lesion classification						
A, (%)	3 (0.8)	0 (0.0)	0.465	1 (1.0)	0 (0.0)	0.957
B1, (%)	25 (6.4)	14 (9.3)		2 (1.9)	3 (2.9)	
B2, (%)	41 (10.4)	15 (10.0)		8 (7.8)	6 (5.8)	
C, (%)	325 (82.5)	121 (80.7)		92 (89.3)	94 (91.3)	
MVD, (%)	313 (79.4)	123 (82.0)	0.504	83 (80.6)	86 (83.5)	0.728
IVUS (%)	172 (43.7)	81 (54.0)	0.031	52 (50.5)	57 (55.3)	0.560
Mean stent diameter, mm	3.0 ± 0.4	3.0 ± 0.4	0.137	3.1 ± 0.4	3.1 ± 0.4	0.988
Total number of stents	2.3 ± 1.1	2.5 ± 1.3	0.221	2.5 ± 1.3	2.5 ± 1.2	0.660
Total stent length, mm	68.5 ± 34.0	70.3 ± 39.5	0.636	70.5 ± 36.8	70.5 ± 36.5	0.963
Procedure success (%)	385 (97.7)	140 (93.3)	0.013	103 (100.0)	100 (97.1)	0.250

MVD, multivessel disease; IVUS, intravascular ultrasound sonography.

**Table 4:** Clinical outcomes.

	Overall						Propensity Score Matching					
	High	Moderate/Low		Multivariate **			High	Moderate/Low		Multivariate **		
	*n* = 394	*n* = 150	log-Rank *p*-Value	HR	95%CI	*p*-Value	*n* = 103	*n* = 103	log-Rank *p*-Value	HR	95%CI	*p*-Value
Target vessel failure	44 (11.2)	19 (12.7)	0.501	0.952	0.539–1.683	0.867	19 (18.5)	12 (11.7)	0.266	0.666	0.323–1.371	0.270
All-cause death	26 (6.6)	7 (4.7)	0.437	0.702	0.290–1.698	0.432	10 (9.7)	5 (4.9)	0.214	0.513	0.175–1.500	0.222
Cardiac death	20 (5.1)	4 (2.7)	0.247	0.439	0.141–1.373	0.157	9 (8.7)	3 (2.9)	0.091	0.342	0.093–1.264	0.108
MI	11 (2.8)	5 (3.3)	0.679	1.144	0.377–3.475	0.812	2 (1.9)	2 (1.9)	0.942	1.075	0.151–7.639	0.942
Target vessel MI	5 (1.3)	2 (1.3)	0.921	0.887	0.154–5.115	0.894	1 (1.0)	0 (0.0)	0.323	-	-	-
Any revascularization	34 (8.6)	16 (10.7)	0.379	1.063	0.575–1.966	0.845	13 (12.6)	10 (9.7)	0.627	0.815	0.358–1.860	0.628
Target vessel revascularization	25 (6.4)	13 (8.7)	0.283	1.146	0.569–2.311	0.703	11 (10.7)	8 (7.8)	0.566	0.767	0.308–1.906	0.568
Target lesion revascularization	21 (5.3)	10 (6.7)	0.476	0.967	0.437–2.137	0.933	9 (8.7)	5 (4.9)	0.318	0.577	0.193–1.722	0.324
Non-target lesion revascularization	13 (3.3)	6 (4.0)	0.634	1.198	0.429–3.345	0.730	3 (2.9)	4 (3.9)	0.580	1.521	0.340–6.800	0.583
CVA	6 (1.5)	4 (2.7)	0.369	1.637	0.404–6.632	0.490	1 (1.0)	0 (0.0)	0.551	2.045	0.185–22.549	0.559
Stent thrombosis	3 (0.8)	2 (1.3)	0.525	0.914	0.116–7.232	0.932	1 (1.0)	1 (1.0)	0.970	1.054	0.066–16.869	0.970
Total Bleeding	21(5.3)	9 (6.0)	0.724	1.255	0.559–2.820	0.582	4 (3.9)	6 (5.8)	0.468	1.592	0.449–5.643	0.472
Minor Bleeding	15 (3.8)	3 (2.0)	0.320	0.494	0.139–1.752	0.275	3 (2.9)	3 (2.9)	0.938	1.066	0.215–5.285	0.938
Major Bleeding	6 (1.5)	6 (4.0)	0.076	3.775	1.095–13.011	0.035	1 (1.0)	3 (2.9)	0.293	3.162	0.329–30.420	0.319

** adjusted by age, sex, body mass index (BMI), systolic blood pressure (SBP), heart failure, serum albumin, triglyceride, dual antiplatelet therapy (DAPT), beta blockers, intravascular ultrasound sonography (IVUS), procedure success. MI, myocardial infarction; CVA, cerebrovascular accident.

## Data Availability

The data included in this manuscript cannot be shared publicly, due to the need to protect the privacy of the included subjects. Data may be shared upon reasonable request to the corresponding author.
